# Pharmacophore-Based Virtual Screening for Identification of Adenosine Deaminase Inhibitors

**DOI:** 10.5812/ijpr-170515

**Published:** 2026-07-20

**Authors:** Saba Ahmadi, Mona Khoramjouy, Arefeh Jafarian, Soraya Shahhosseini, Salimeh Amidi, Farzad Kobarfard

**Affiliations:** 1 Department of Medicinal Chemistry, School of Pharmacy, Shahid Beheshti University of Medical Sciences, Tehran, Iran; 2 Phytochemistry Research Center, Shahid Beheshti University of Medical Sciences, Tehran, Iran; 3 Research Center for War-Affected People, Tehran University of Medical Sciences, Tehran, Iran; 4 Central Research Laboratories, Shahid Beheshti University of Medical Sciences, Tehran, Iran

**Keywords:** Adenosine Deaminase, Pharmacophore Modeling, Molecular Docking, Hits, Virtual Screening, Enzyme Inhibition

## Abstract

**Background:**

Adenosine deaminase (ADA) is a key enzyme in purine metabolism, and abnormal ADA activity has been associated with various diseases, including severe combined immunodeficiency, cancer, neurodegenerative disorders, and liver diseases.

**Objectives:**

This study employed a pharmacophore-based virtual screening strategy to identify novel ADA inhibitors from an in-house library.

**Methods:**

Two crystal structures of ADA co-crystallized with erythro-9-(2-hydroxy-3-nonyl) adenine (EHNA) and pentostatin were used to develop a pharmacophore model. The validated model was used to screen an in-house library. The resulting hits were further evaluated using molecular docking with AutoDock Vina. The compound with the highest binding affinity in the docking study was subsequently assessed by molecular dynamics (MD) simulation and in silico absorption, distribution, metabolism, excretion, and toxicity (ADMET) analysis.

**Results:**

The validated pharmacophore model comprised one hydrogen bond donor, one hydrogen bond acceptor, and one aromatic ring. When evaluated against the DUDE-Z database set, the model demonstrated acceptable sensitivity and specificity, with an enrichment factor of 23. Screening of the in-house library identified four promising hits. Compound 154 was the most notable hit because of its potent binding affinity and favorable interactions with key amino acids in the ADA active site. MD simulations showed that the ADA–compound 154 complex remained stable throughout the 150 ns simulation. MM-GBSA energy analysis indicated favorable binding driven by van der Waals and electrostatic interactions. Per-residue decomposition analysis identified critical residues contributing to complex stabilization. Compared with reference ADA inhibitors, compound 154 exhibited similar drug-likeness and pharmacokinetic properties, although further toxicity optimization may be required.

**Conclusions:**

These results suggest that compound 154 is a promising lead for developing new ADA inhibitors and underscore the effectiveness of computational methods in drug discovery.

## 1. Background

Adenosine deaminase (ADA), also known as adenosine dehydrolyase, is the principal enzyme responsible for purine metabolism and catalyzes the deamination of adenosine and 2'-deoxyadenosine to inosine and deoxyinosine, respectively (1, 2).

This enzyme is present in human and mammalian tissues, particularly lymphoid tissues. Its expression level is important in physiological and pathological processes (3). Several studies have indicated that elevated ADA levels play a significant role in the pathogenesis of various cancers. ADA overactivity is also implicated in AIDS, Parkinson disease, and liver disorders, such as chronic hepatitis, cirrhosis, and hepatocellular carcinoma (4-6). Conversely, ADA deficiency is associated with several disorders, particularly severe combined immunodeficiency, a rare and life-threatening genetic disease. In severe combined immunodeficiency, genetic mutations affecting ADA activity impair the proliferation and differentiation of B and T lymphocytes, leading to immune system dysfunction and increased susceptibility to serious infections (7-9).

**Figure 1.**
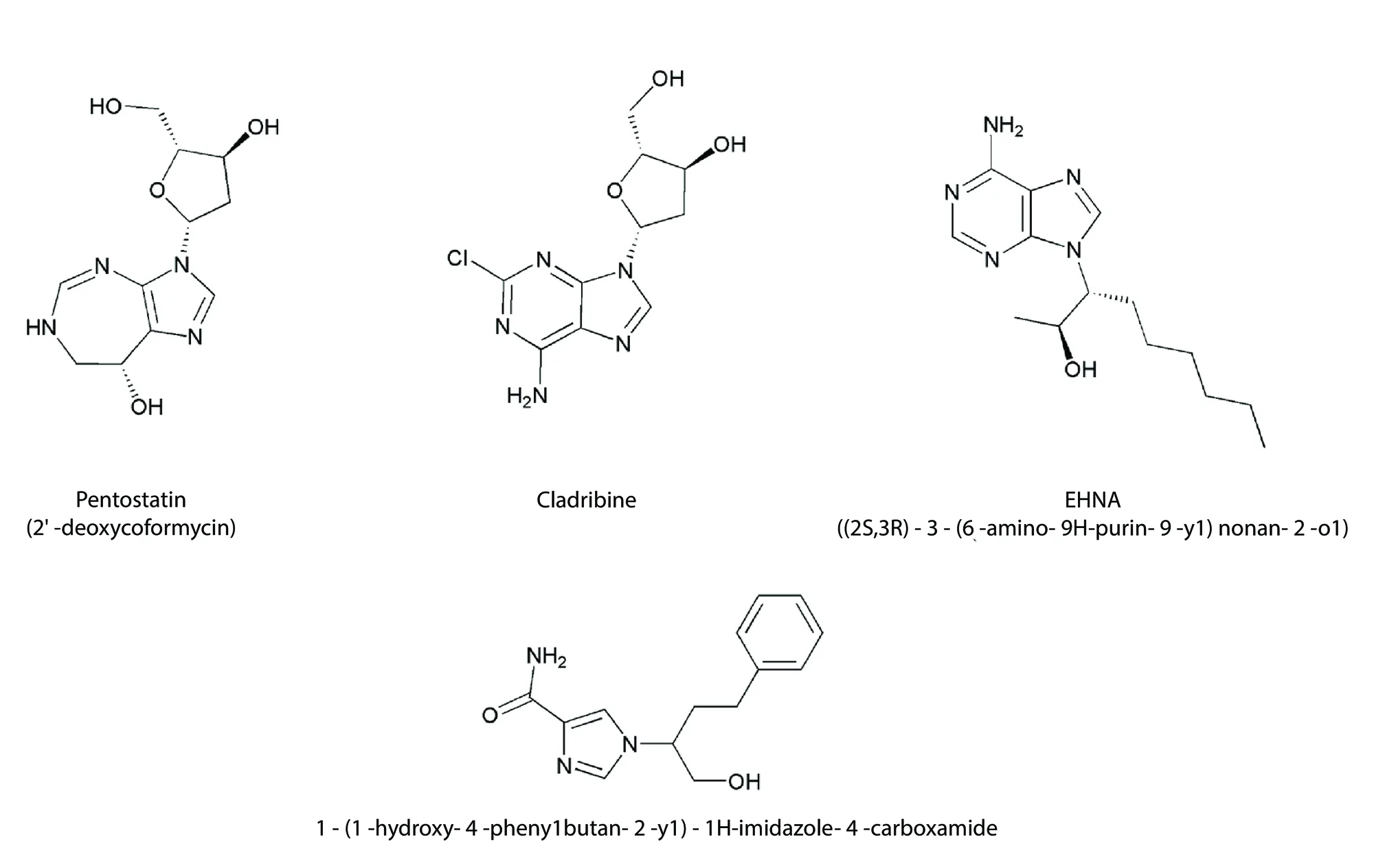
Structure of selected ADA inhibitors. Pentostatin and cladribine are nucleoside-based inhibitors, EHNA is an adenine-like inhibitor, and 1-(1-hydroxy-4-phenylbutan-2-yl)-1H-imidazole-4-carboxamide is a non-nucleoside inhibitor.

ADA is a metalloenzyme comprising a (β/α)_8_-barrel structure with a zinc ion in the active site (2, 10). Various chemical and phytochemical compounds, such as flavonoids, have been identified as potential inhibitors of ADA activity. Currently, pentostatin and cladribine are the two FDA-approved ADA inhibitors. However, other potent inhibitors, such as EHNA, failed to enter clinical trials because of poor pharmacokinetic properties (Figure 1) (2). Known ADA inhibitors may contain a nucleoside framework, such as ribose and a nucleobase, as in pentostatin, or adenine-derived scaffolds linked to a hydrophobic chain, as in EHNA. Additionally, ADA inhibitors may have non-nucleoside structures, such as quercetin and 1-(1-hydroxy-4-phenylbutan-2-yl)-1H-imidazole-4-carboxamide (1, 10-14). The identification and development of novel ADA inhibitors with therapeutic potential in various diseases, such as cancer, neurodegenerative disorders, and inflammatory conditions, remain major challenges in drug discovery (15).

New targets and hits are discovered daily using traditional methods, which are time-consuming and expensive. As an alternative, computer-aided drug discovery (CADD) methods offer cost-effective and rapid approaches to drug discovery. Pharmacophore-based techniques, as part of CADD, can be used for virtual screening, lead optimization, hit identification, and multitarget drug design. Virtual screening is a computational approach that screens large compound libraries to identify potential hits (16, 17).

Pharmacophore modeling is divided into two main categories: structure-based and ligand-based (18). In the structure-based approach, structural information about the target, such as enzymes or receptors, is used, whereas the ligand-based approach relies on developing 3D pharmacophore models and quantitative structure-activity/property relationship models derived from the physicochemical properties of known ligands to identify hits (17, 18).

## 2. Objectives

The aim of this study was to develop and validate a structure-based pharmacophore model to screen an in-house library for potential ADA inhibitors. Molecular docking studies and MD simulations were performed to evaluate the interactions and stability of the selected hit compounds with the target enzyme. In addition, in silico analysis of ADMET properties was conducted.

## 3. Methods

### 3.1. Pharmacophore Model Generation

The crystallographic structures of ADA from Plasmodium vivax co-crystallized with pentostatin (PDB ID: 2PGR) and ADA from *Bos taurus* co-crystallized with EHNA (PDB ID: 2Z7G) were downloaded from the RCSB Protein Data Bank (https://www.rcsb.org) (19, 20).

For structure-based pharmacophore model generation, the selected PDB structures were directly imported into LigandScout 4.5. Because the ADA protein contains a single chain (chain A) encompassing the active site, this chain was used for analysis. Crystallographic water molecules involved in key ligand–protein interactions, as well as the zinc ion, were retained. The default LigandScout settings were used for feature detection, excluded-volume generation, and distance constraints, without manual modification of any parameters. The generated pharmacophore models were subsequently aligned using the built-in alignment tool in LigandScout to identify common pharmacophoric features (21). *Multiple pharmacophore* models were independently generated from the 2Z7G and 2PGR crystal structures and refined by adding or removing pharmacophoric features to optimize model performance.

### 3.2. Pharmacophore Model Validation

The generated pharmacophore models were evaluated using a dataset obtained from the DUDE-Z database (https://dudez.docking.org) for ADA, consisting of 66 known active compounds and 3300 decoys (22-24). The validation set contained various classes of ADA inhibitors, including nucleoside-based inhibitors, adenine-derived scaffolds, and non-nucleoside inhibitors.

The pharmacophore model was validated using sensitivity (Se), specificity (Sp), accuracy (Acc), yield of actives (Ya), goodness-of-hit (GH) score, enrichment factor (EF), receiver operating characteristic (ROC) curve analysis, and the corresponding area under the curve (AUC) (25, 26).

Sensitivity, also referred to as recall, reflects the ability of the model to correctly identify true-positive compounds, whereas specificity represents the ability of the model to correctly identify true-negative compounds. Accuracy describes the overall predictive performance of the model by considering both true-positive and true-negative predictions. Yield of actives represents the ability of the model to determine the fraction of true positives among the retrieved hits. The GH score combines Se, Sp, and Ya to evaluate the overall performance of the pharmacophore model.

The values of Se, Sp, Acc, Ya, and GH range from 0 to 1, with higher values indicating better model performance. GH values between 0.4 and 0.6 are generally considered moderately predictive and suitable for virtual screening.

The enrichment factor is defined as the ratio of true positives in the hit list to active compounds in the entire database and evaluates the model’s ability to retrieve active compounds relative to random selection. Higher EF values indicate better early recognition capability.

ROC curve analysis was performed by plotting sensitivity against 1 - specificity to assess the discriminatory power of the pharmacophore model. The AUC was used as an additional measure of model performance. An ideal ROC curve would yield an AUC of 1, whereas an AUC value below 0.5 indicates performance worse than random selection (25-27).

### 3.3. Virtual Screening

The best-validated pharmacophore model was used to virtually screen an in-house library consisting of 155 compounds, including previously synthesized compounds and newly designed structures. These compounds were originally developed for various targets and were screened in this study as potential ADA inhibitors.

The in-house library showed considerable structural diversity and a broad range of physicochemical properties. The compounds had molecular weights ranging from 118 to 490 g/mol, lipophilicity (cLogP) between -2 and 5, hydrogen bond donor counts ranging from 0 to 3, hydrogen bond acceptor counts between 1 and 12, and topological polar surface area (TPSA) values ranging from 12 to 193 Å^2^.

The in-house library compounds were prepared in SDF format and imported into LigandScout. Virtual screening was performed using default settings, and hits were ranked based on their pharmacophore-fit scores. Compounds exhibiting high fit scores and acceptable feature matching were selected as potential hits for subsequent molecular docking analysis.

### 3.4. Docking Studies

Molecular docking studies were performed for the selected hits obtained from virtual screening. The crystal structures (PDB IDs: 2Z7G and 2PGR) were used as target proteins, and the centers of the co-crystallized ligands were used as the centers of the grid boxes. A cubic grid box was used with dimensions of 20 × 20 × 20 Å^3^. The grid box centers were defined as center x = 29.61, y = 23.67, and z = 37.43 for 2PGR and center x = 10.75, y = 15.60, and z = 20.97 for 2Z7G.

Hyperchem (version 8.0) was used to draw the ligands. The Molecular Mechanics force field (MM^+^) was used for energy minimization. The proteins were prepared according to previously reported protocols (28). Briefly, water molecules and ligands in the crystal structures of the proteins were removed. All hydrogens were added to the proteins, and nonpolar hydrogens were merged using AutoDock Tools (version 1.5.6rc3). Kollman charges were assigned to the proteins, and the structures were saved in PDBQT format. Ligands were also converted to PDBQT format using the same tool.

Docking was performed using AutoDock Vina (version 1.2.0) with an exhaustiveness value of 100, an energy range of 3 kcal/mol, and a maximum of 10 output poses. The best binding poses were selected based on binding affinity (kcal/mol) and analyzed for ligand–protein interactions using Discovery Studio Visualizer (version 4.5). Double bonds in the molecular structures were corrected where necessary. Hydrophobic interactions, including π-alkyl, aromatic hydrophobic, and alkyl hydrophobic interactions with distances below 5 Å, as well as hydrogen bonds with donor–acceptor distances below 3.5 Å, were considered significant and reported.

The docking protocol was validated by redocking the co-crystallized ligands into the active site. PyMOL (version 0.99rc6) was used to calculate the root mean square deviation (RMSD) between the predicted and experimental poses.

### 3.5. Molecular Dynamics Simulation

Molecular dynamics simulations were conducted using GROMACS 5.1.4 software (29). The protein was parameterized using the CHARMM36 force field. Ligand topologies were prepared using the CHARMM General Force Field. Ligand structures were submitted to the CGenFF server for automated topology and parameter generation. Ligands were complexed with the target protein and placed in a cubic box of the TIP3P water model, maintaining a minimum distance of 1.0 nm between the solute and the box boundaries. To neutralize the system, sodium (Na^+^) and chloride (Cl^-^) ions were added as counterions to achieve an ionic concentration of 0.15 M. Energy minimization was carried out using the steepest descent algorithm until the convergence criteria were reached. The minimized system underwent two sequential equilibration phases: the NVT ensemble followed by the NPT ensemble. NVT was performed for 100 ps under a constant number of particles and volume at 300 K using a modified Berendsen thermostat for temperature coupling. NPT was performed for an additional 100 ps at 300 K and 1 bar using the Parrinello-Rahman barostat for pressure coupling. A 2 fs time step was applied in both phases.

A 150 ns unrestrained production MD simulation was performed under NPT conditions on a multicore computing environment. The leap-frog integrator was used with a 2 fs time step, and periodic boundary conditions were applied in all directions. The particle mesh Ewald method was used to handle long-range electrostatic interactions, whereas a 1.0 nm cutoff was used to compute short-range electrostatic and van der Waals interactions. The LINCS algorithm was applied to constrain all covalent bonds involving hydrogen atoms (30).

The structural stability and dynamic behavior of the protein without a ligand and of the ligand-bound complexes were evaluated through standard analyses, including RMSD, radius of gyration (Rg), root mean square fluctuation (RMSF), and hydrogen bond (H-bond) analysis (30). For both protein and ligand RMSD analyses, the initial minimized and equilibrated structure was used as the reference to monitor deviation from the starting simulated conformation. Before RMSD calculation, all trajectory frames were least-squares fitted using the protein backbone atoms (N, Cα, and C). Protein RMSD values were then calculated using the same backbone atoms relative to the reference structure. For ligand RMSD analysis, the protein backbone alignment obtained from the fitting procedure was maintained, and RMSD values were calculated using the ligand heavy atoms (all nonhydrogen atoms) relative to the reference ligand coordinates.

### 3.6. MM-GBSA and Per-Residue Decomposition Energy Calculations

To quantify the thermodynamic stability of the complex, binding free energy (ΔG_bind_) was calculated using the MM-GBSA method based on the last 50 ns of simulation, with sampling at 2 ps intervals, using the gmx_mmpbsa_ tool. The total binding free energy was calculated according to the following equations (31):

Equation 1.

$$∆G_{\mathrm{bind}}=∆G_{\mathrm{GAS}}+∆G_{\mathrm{SOLV}}$$

Equation 2.

$$∆G_{\mathrm{GAS}}=∆E_{\mathrm{EL}}+∆E_{\mathrm{VDWAALS}}$$

Equation 3.

$$∆G_{\mathrm{SOLV}}=∆G_{\mathrm{GB}}+∆G_{\mathrm{SURF}}$$

where ΔE_VDWAALS_ and ΔE_EL_ represent van der Waals and electrostatic interactions, respectively, while ΔG_GB_ and ΔG_SURF_ correspond to polar and nonpolar solvation energies. ΔG_GAS_ is gas-phase energy, and ΔG_SOLV_ is solvation free energy (32). In addition, per-residue decomposition energy analysis was performed to identify key residues that play significant roles in the overall binding affinity and stability of the complex (30).

### 3.7. In Silico Physicochemical and ADMET Properties

The physicochemical and ADMET-related properties of the selected compounds were predicted using the ADMETlab 3.0 server (https://admetlab3.scbdd.com) (33). The SMILES formats of the compounds were imported into the server, and various physicochemical, pharmacokinetic, drug-likeness, and toxicity-related parameters were predicted.

## 4. Results and Discussion

### 4.1. Pharmacophore Modeling

The crystal structures of ADA (PDB IDs: 2Z7G and 2PGR), co-crystallized with two well-known potent inhibitors, EHNA (an adenine-like inhibitor) and pentostatin (a nucleoside-based inhibitor), were selected for pharmacophore modeling to capture the key pharmacophoric features of the major inhibitor classes.

**Figure 2.**
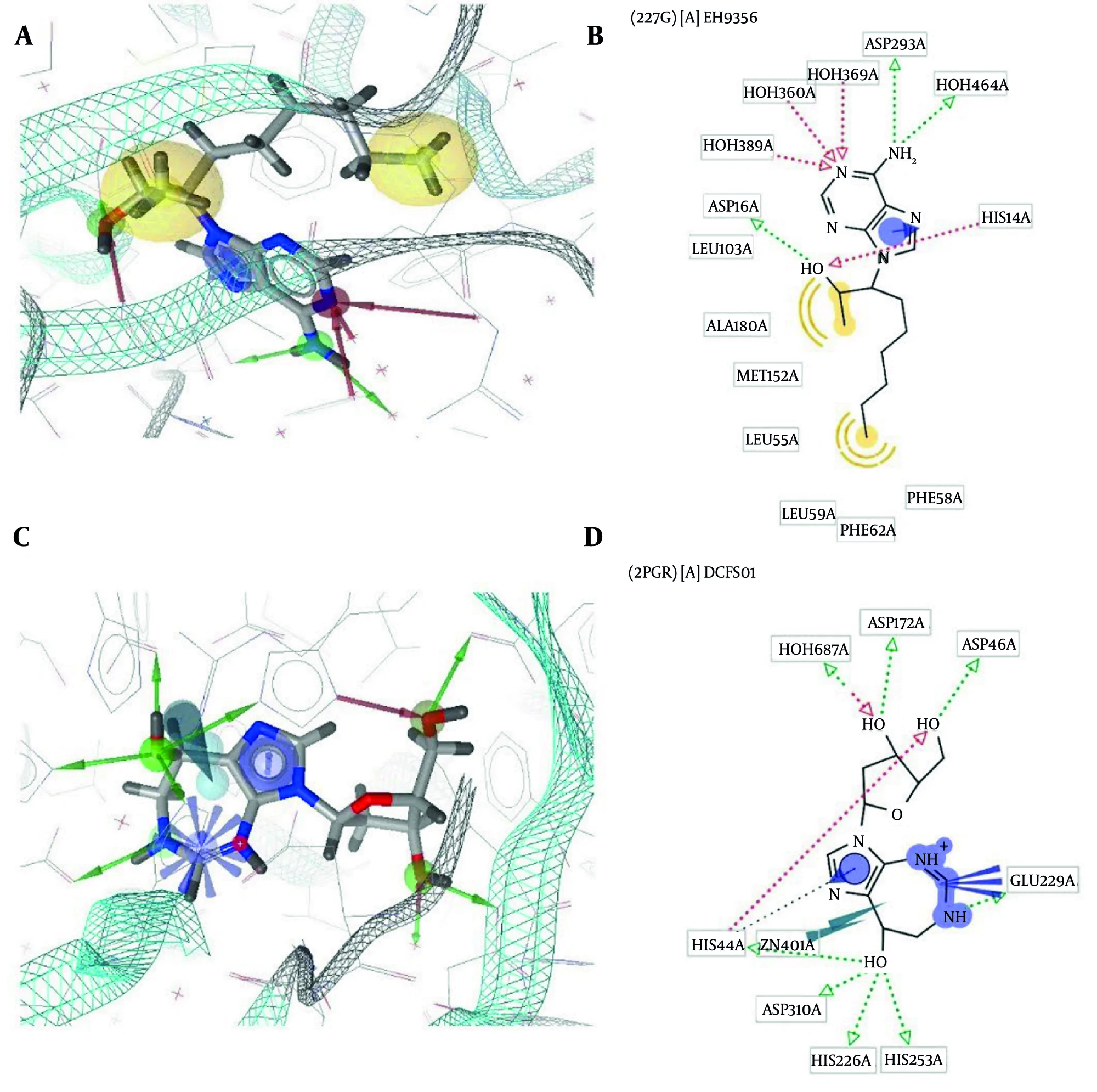
Structure-based (A) and 2D diagram (B) of the pharmacophore model of 2Z7G-EHNA and structure-based (C) and 2D diagram (D) of the pharmacophore model of 2PGR-pentostatin.

The pharmacophore model generated from 2Z7G (EHNA-bound) comprised three hydrogen bond donors, four hydrogen bond acceptors, one aromatic ring, and two hydrophobic features. In contrast, the model derived from 2PGR (pentostatin-bound) comprised eight hydrogen bond donors, two hydrogen bond acceptors, one aromatic ring, one positive ionizable center, and two zinc-binding features (Figure 2).

Multiple pharmacophore models were also generated from each ADA–ligand complex and evaluated based on their ability to discriminate active compounds from decoys using sensitivity. Models 1 to 5 are presented in Table 1.

**Table 1.** Pharmacophoric Features of 2Z7G, 2PGR, and the Shared-Feature Pharmacophore Model and Model Se ^a^

Model	Description	HBD	HBA	H	AR	PI	ZNB	hits	TP	FP	Se
**1**	PDB ID: 2Z7G	3	4	2	1	-	-	8	8	0	0.12
**2**	PDB ID: 2PGR	8	2	-	1	1	2	0	-	-	-
**3**	MF	10	5	2	1	1	1	0	-	-	-
**4**	F	2	1	-	1	-	-	47	15	32	0.23
**5**	SF	1	1	-	1	-	-	87	40	47	0.61

^a^ Abbreviations: HBD, hydrogen bond donor; HBA, hydrogen bond acceptor; H, hydrophobic; AR, aromatic ring; PI, positive ionization area; ZNB, zinc-binding location; TP, true positive; FP, false positive; Se, sensitivity.

A shared-feature (SF) pharmacophore model (model 5) was selected because it demonstrated better performance than the other models. The SF pharmacophore model consists of one hydrogen bond donor, one hydrogen bond acceptor, and one aromatic ring feature. The distance between the hydrogen bond donor and hydrogen bond acceptor features was 7.33 Å, whereas the distances between the hydrogen bond donor–aromatic ring and hydrogen bond acceptor–aromatic ring features were 3.46 Å and 4.44 Å, respectively. The tolerance radii for each feature were set according to the default settings of LigandScout, ranging between 1.0 and 1.5 Å and allowing flexibility in ligand mapping. In addition, the relative orientation of the features was evaluated, and the angles formed between the three pharmacophoric points (hydrogen bond donor-hydrogen bond acceptor-aromatic ring) were 24.75°, 19.04°, and 136.21°.

**Figure 3.**
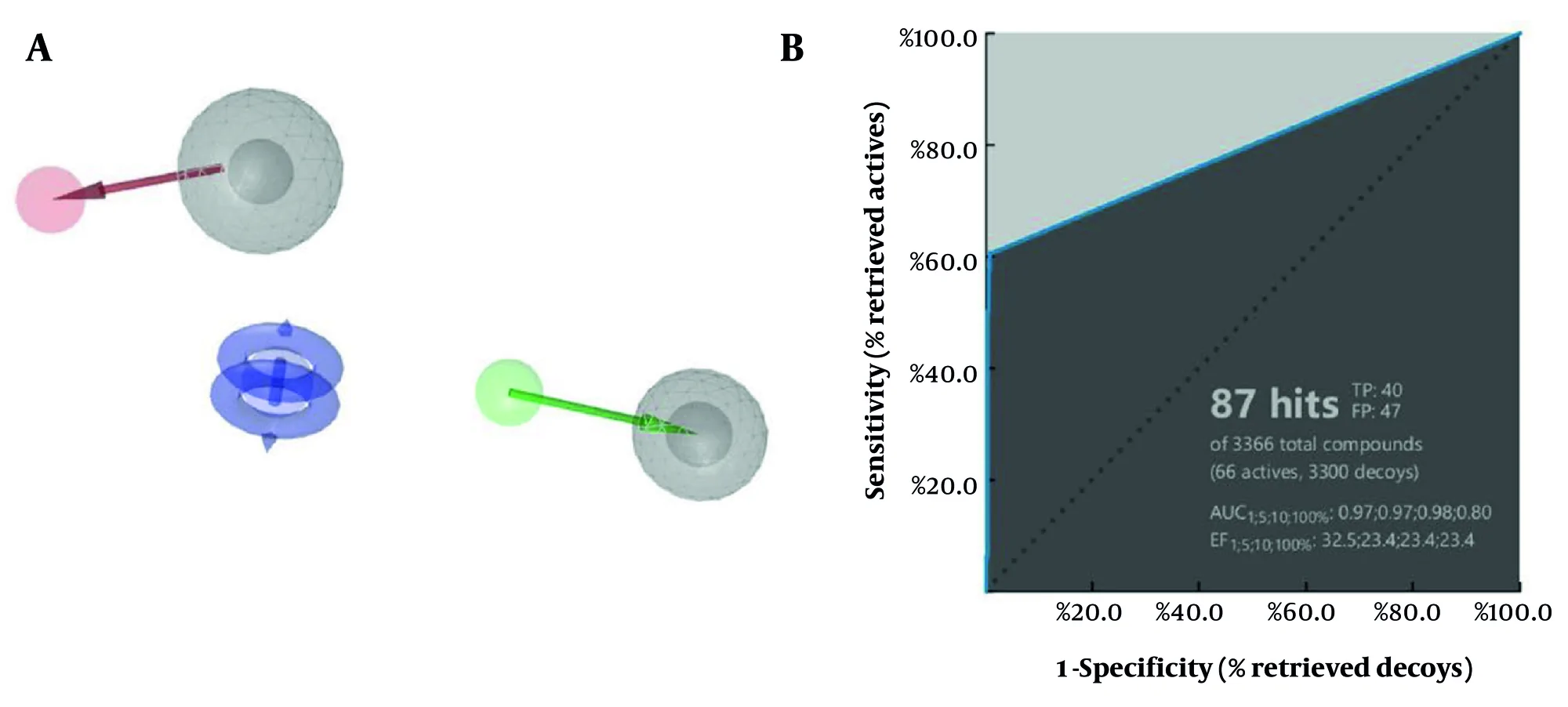
Pharmacophore model with exclusion volumes; hydrogen bond acceptor (HBA) in red, hydrogen bond donor (HBD) in green, aromatic ring (AR) in blue, and exclusion volumes in gray. The distances (Å) between pharmacophoric features are 7.33 Å (HBD-HBA), 3.46 Å (HBD-AR), and 4.44 Å (HBA-AR). The angles formed between the three pharmacophoric points (HBD-HBA-AR) were 24.75°, 19.04°, and 136.21° (A). Receiver operating characteristic curve generated for the pharmacophore model (B).

The performance of the SF model was evaluated using a dataset consisting of 66 known active compounds and 3300 decoy molecules (total D = 3366) obtained from the DUDE-Z database for ADA. The validation parameters of the SF model are presented in Table 2 and Figure 3.

**Table 2.** Validation Parameters of the SF Model ^a^

Parameters	SF Model
**Total molecules in database (D)**	3366
**Total number of actives in database (P)**	66
**Total number of inactive compounds in database (N)**	3300
**Total hits (n)**	87
**TP**	40
**TN**	3253
**FP**	47
**FN**	26
**Se (TP/TP + FN)**	0.61
**Sp (TN/TN + FP)**	0.98
**Acc ((TP + TN)/(P + N))**	0.97
**Yield of actives (TP/n)**	0.46
**EF ((TP × D)/(n × P))**	23.4
**GH score**	0.49

^a^ Abbreviations: Se, Sensitivity; Sp, specificity; Acc, accuracy; EF, enrichment factor; GH, goodness of hit; TP, true positives; TN, true negatives; FP, false positives; FN, false negatives.

The SF model had a sensitivity of 0.60, specificity of 0.98, accuracy of 0.97, and an enrichment factor of 23.4. These results indicated that the model has a strong ability to distinguish active compounds from decoys. The GH score of 0.49 indicates medium quality, which is considered acceptable for virtual screening. The model demonstrated excellent early recognition capability, with AUC values of 0.97, 0.97, and 0.98 at 1%, 5%, and 10% of the screened database, respectively. Moreover, the total AUC value of 0.80 demonstrates the good discriminatory ability of the selected pharmacophore model. The enrichment factor of the SF model indicated excellent early enrichment performance, with EF values of 32.5, 23.4, and 23.4 at 1%, 5%, and 10% of the screened database, respectively. These EF values indicate that the pharmacophore model is highly effective at ranking active compounds. Collectively, these results demonstrate that the SF model is accurate and practical for virtual screening of the in-house library to identify potential ADA inhibitors.

### 4.2. Virtual Screening

**Figure 4.**
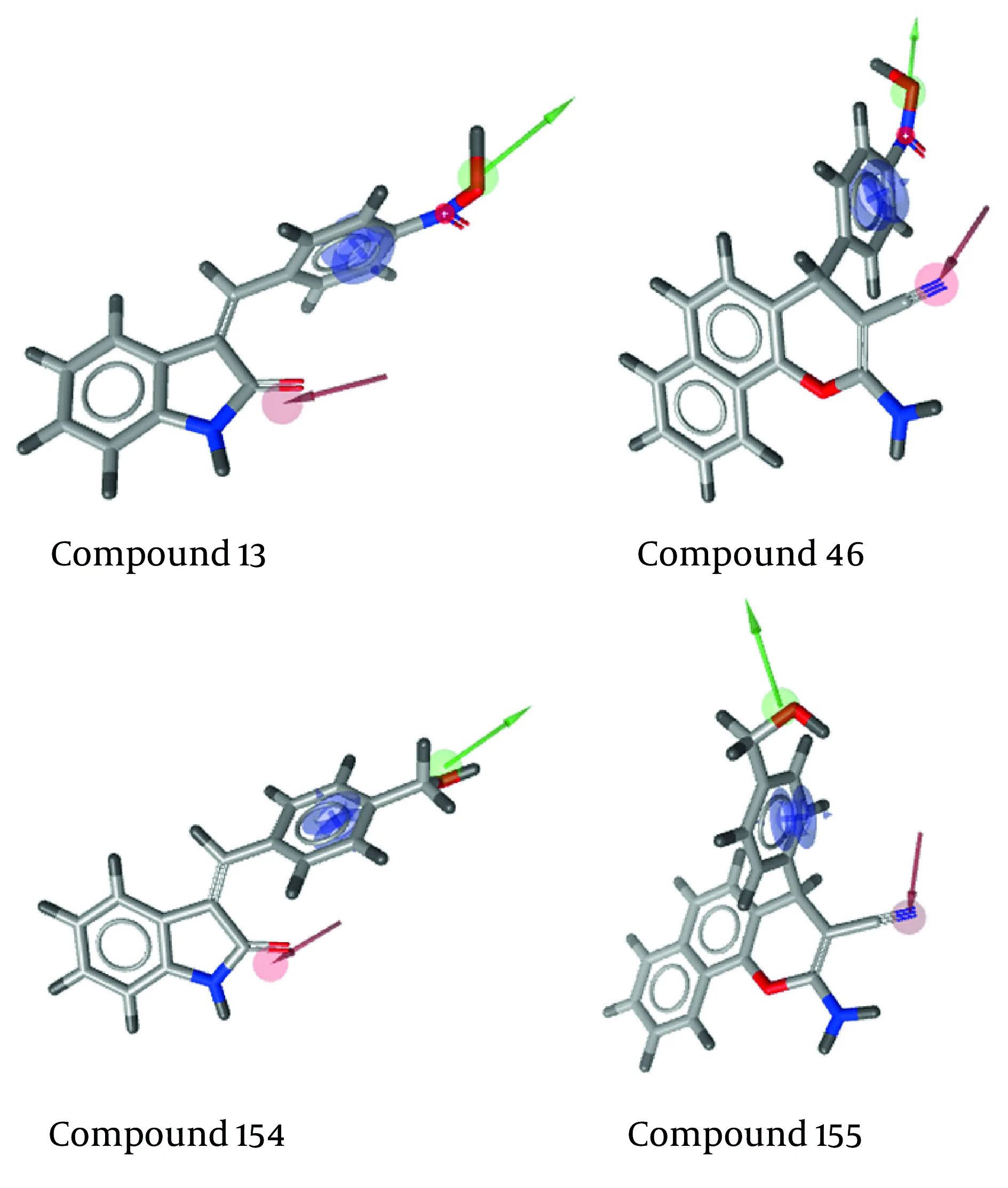
Obtained hit compounds aligned to the 3D pharmacophore model (SF model).

The in-house library comprising 155 compounds was screened using the validated SF model. Four hit compounds, 13, 46, 154, and 155, were identified based on pharmacophore-fit scores and their ability to match the three features of the pharmacophore model (Figure 4). The identified hit compounds had non-nucleoside structures. However, further biochemical and kinetic studies are required to confirm their inhibitory mechanisms.

### 4.3. Molecular Docking Studies

Molecular docking studies of the four hit compounds identified by virtual screening were performed using AutoDock Vina. The crystal structures of ADA (PDB IDs: 2Z7G and 2PGR), co-crystallized with EHNA and pentostatin, respectively, were used for molecular docking. The RMSD values obtained from redocking of the co-crystallized ligands for both 2Z7G and 2PGR were below 2 Å, confirming the validity of the docking procedure. The binding affinities of the co-crystallized ligands and hit compounds are summarized in Table 3.

**Table 3.** Binding Affinities (kcal/mol) of Hit Compounds with Adenosine Deaminase

Compounds ID	Name of the Compounds	PDB ID: 2Z7G	PDB ID: 2PGR
**13**	(Z)-3-(4-nitrobenzylidene)indolin-2-one	-9.3	-8.5
**46**	(S)-2-amino-4-(4-nitrophenyl)-4H-benzo[h]chromene-3-carbonitrile	-10.3	2.0
**154**	(Z)-3-(4-(hydroxymethyl)benzylidene)indolin-2-one	-9.0	-9.1
**155**	(R)-2-amino-4-(4-(hydroxymethyl)phenyl)-4H-benzo[h]chromene-3-carbonitrile	-9.6	-2.5
**EHNA ^a^**	Erythro-9-(2-hydroxy-3-nonyl)adenine	-6.8	-
**DCF ^b^**	2'-deoxycoformycin (pentostatin)	-	-6.3

^a^ Co-crystallized ligand in PDB: 2Z7G.

^b^ co-crystallized ligand in PDB: 2PGR.

Compounds 46 and 155 showed suitable binding affinity toward 2Z7G, whereas compounds 13 and 154 exhibited favorable binding affinities toward both ADA crystal structures (2Z7G and 2PGR). The differences in binding affinities of compounds 46 and 155 between the two ADA crystal structures can be attributed to structural differences in the co-crystallized ligands and ligand-induced conformational changes within the active site (34).

**Figure 5.**
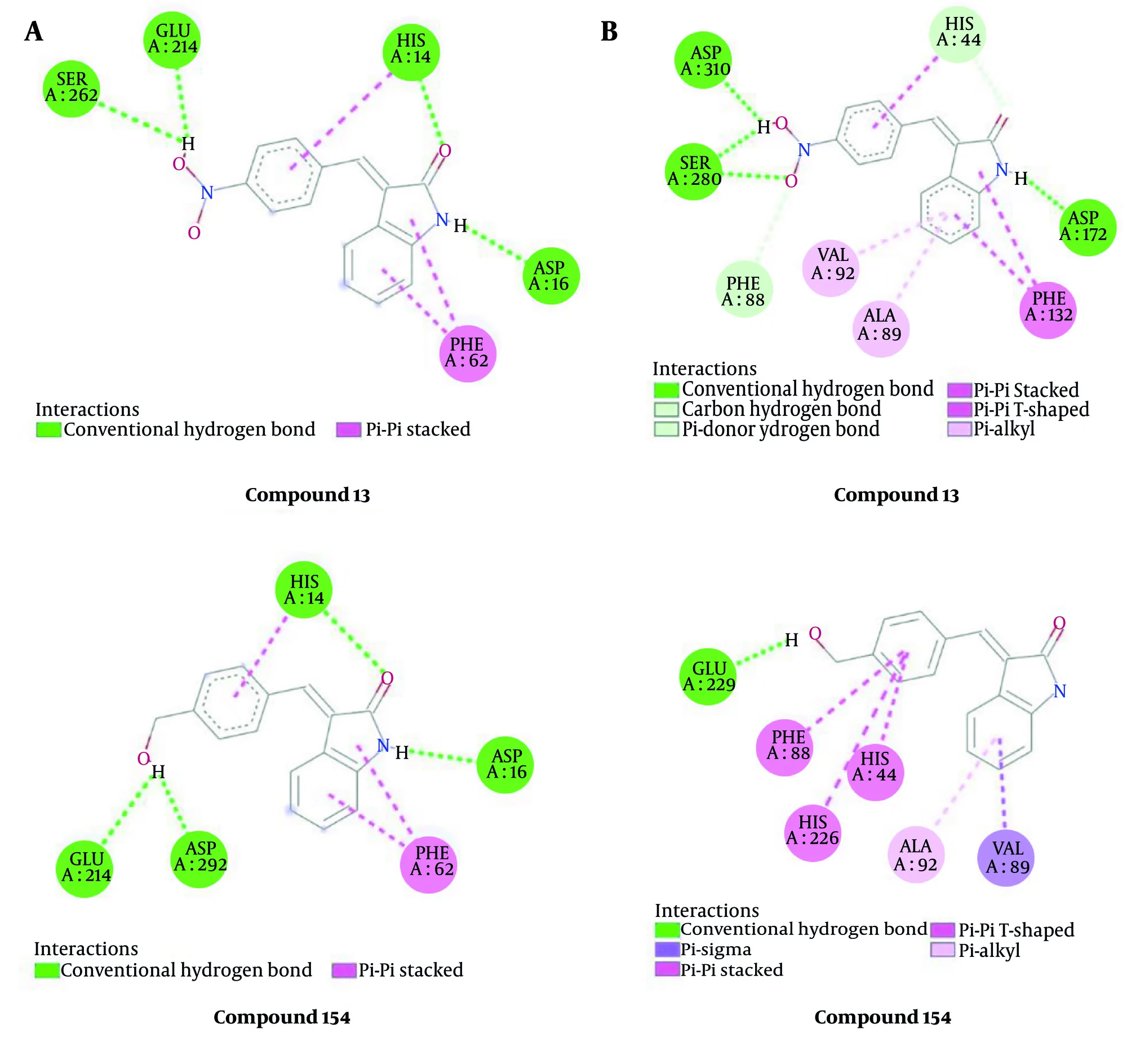
2D interaction between ligands and the active site of ADA (PDB ID: 2Z7G (A) and 2PGR (B).

Based on these results, compounds 13 and 154 demonstrated better binding affinity toward both 2Z7G and 2PGR and were selected for further interaction analysis (Figure 5).

EHNA, the co-crystallized ligand of 2Z7G, exhibited several key interactions, including hydrogen bonds with HIS14 (3.12 Å), GLU214 (2.49 Å), and ASP16 (2.38 Å), as well as hydrophobic interactions with the PHE62, PHE58, LEU59, ALA180, and HIS14 residues. Compound 13 displayed four hydrogen bonds with HIS14 (2.96 Å), SER262 (2.84 Å), GLU214 (2.24 Å), and ASP16 (2.33 Å), along with hydrophobic interactions with PHE62 and HIS14. Compound 154 showed appropriate binding affinity in docking compared with compound 13 and EHNA. This compound exhibited four hydrogen bonding interactions with HIS14 (2.96 Å), GLU214 (2.88 Å), ASP16 (2.34 Å), and ASP292 (2.10 Å), as well as hydrophobic interactions with PHE62 and HIS14.

Pentostatin, the co-crystallized ligand of 2PGR, formed several key interactions, including five hydrogen bonding interactions with GLU229 (2.51 Å), HIS253 (2.38 Å), HIS226 (3.33 Å), ASP310 (2.63 Å), and ASP311 (2.11 Å), in addition to hydrophobic interactions with PHE88, VAL89, and LEU85. Compound 13 showed a binding affinity of -8.5 kcal/mol and formed hydrogen bonds with ASP310 (2.25 Å), ASP172 (1.96 Å), HIS44 (3.37 Å), and SER280 (1.93 Å), along with hydrophobic interactions with HIS44, PHE132, VAL89, and ALA92. Compound 154 exhibited a hydrogen bond with GLU229 (1.96 Å) and hydrophobic interactions with HIS226, PHE88, HIS44, ALA92, PHE132, and VAL89.

Compound 154 was selected for further analysis (MD simulation and ADMET evaluation) because of its low binding affinities across both crystal structures.

### 4.4. MD Simulations

#### 4.4.1. RMSD Analysis

**Figure 6.**
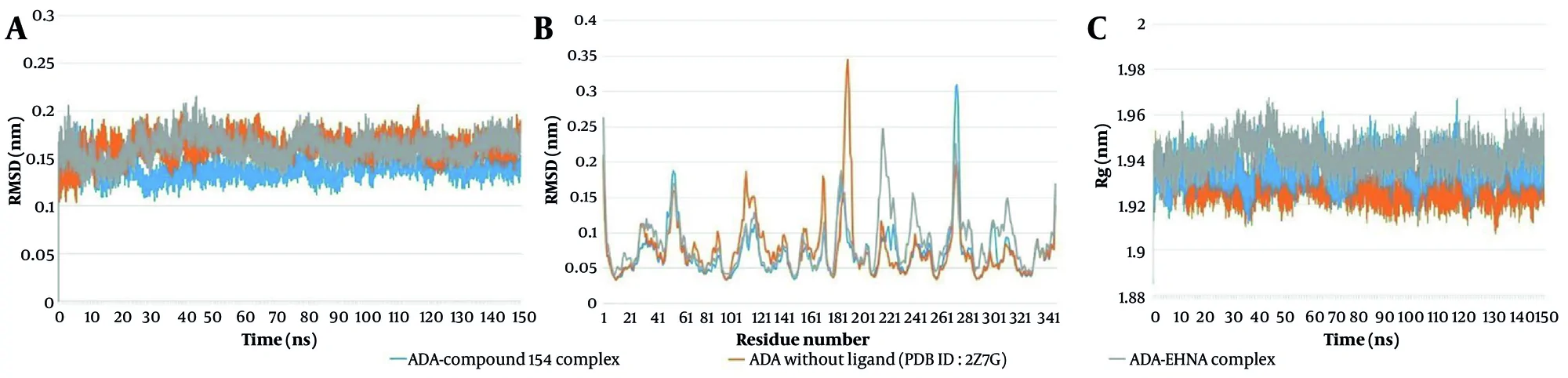
Root mean square deviation (RMSD) (A), root mean square fluctuation (RMSF) (B), and radius of gyration (Rg) (C) analyses of ADA (PDB ID: 2Z7G) without ligand (orange), in complex with EHNA (gray), and in complex with compound 154 (blue) during 150 ns molecular dynamics simulations.

Because compound 154, the selected hit, has a non-nucleoside structure, the ADA crystal structure 2Z7G co-crystallized with EHNA was selected for MD simulations. The structural stability of ADA without a ligand and in complex with EHNA (reference) and compound 154 during the 150 ns MD simulation was evaluated using RMSD analyses. RMSD values provide insight into conformational changes and the stability of the complex during the simulation and were calculated for the complex backbone atoms relative to the initial structure recorded at 0.01 ns intervals. All systems showed relatively stable trajectories with reduced fluctuations throughout the simulation. The ADA–compound 154 complex exhibited the lowest average RMSD value with the smallest fluctuations (0.139 ± 0.0097 nm) compared with ADA without a ligand and the complex with the reference. In addition, during the last 50 ns, the ADA–compound 154 complex remained stable compared with the other systems, with an average RMSD of 0.142 nm. These results indicate structural stability upon ligand binding and suggest that compound 154 contributes to maintaining a more stable and compact protein conformation during the simulation (Figure 6A).

**Figure 7.**
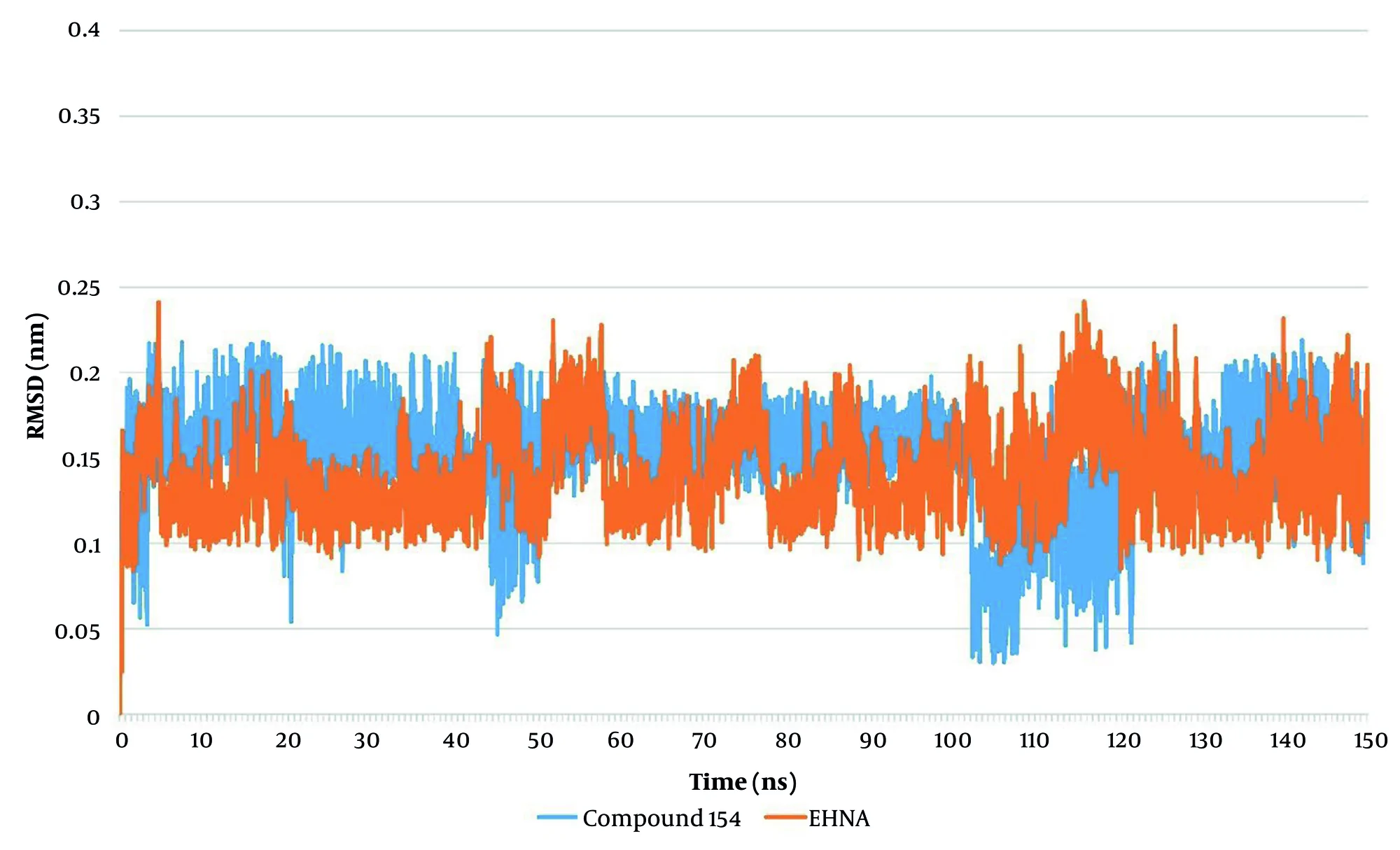
Root mean square deviation profiles of compound 154 (blue) and EHNA (orange) as the reference ligand during the 150 ns molecular dynamics simulation.

The conformational stability of the ligands (compound 154 and EHNA as reference) within the binding pocket was investigated using ligand RMSD analysis over the 150 ns MD simulation. Both ligands exhibited an initial increase in RMSD during the equilibration phase because of adaptation to the binding environment. Compound 154 showed an average RMSD of 0.152 nm, slightly higher than that of the reference ligand (0.140 nm) (Figure 7).

#### 4.4.2. RMSF Analysis

The RMSF profile of the protein systems revealed varying degrees of flexibility across the ADA (2Z7G) residues over the 150 ns MD simulation. The ADA–compound 154 complex exhibited the lowest average RMSF value (0.077 ± 0.039 nm) compared with ADA without a ligand (0.082 ± 0.045 nm) and the complex with the reference (0.089 ± 0.048 nm). The low RMSF values (< 0.1 nm) indicate protein backbone stability with localized flexible regions (Figure 6B).

#### 4.4.3. Gyration Analysis

To evaluate the compactness and overall structural stability of ADA without a ligand and in complex with EHNA (reference) and compound 154, the radius of gyration was analyzed over the 150 ns MD simulation (Figure 6C).

All systems showed stable Rg profiles with minimal fluctuations (standard deviation within ± 0.006 nm), indicating preservation of structural integrity throughout the simulation. The ADA–compound 154 complex exhibited an average Rg value of 1.932 nm, comparable to ADA without a ligand and slightly lower than the ADA–reference complex (1.940 nm). These results suggest a more compact conformation in the presence of compound 154 without disruption of the protein fold. These data support the formation of a stable and compact ligand–protein complex, consistent with the RMSD and RMSF results.

#### 4.4.4. Hydrogen Bond Analysis

**Figure 8.**
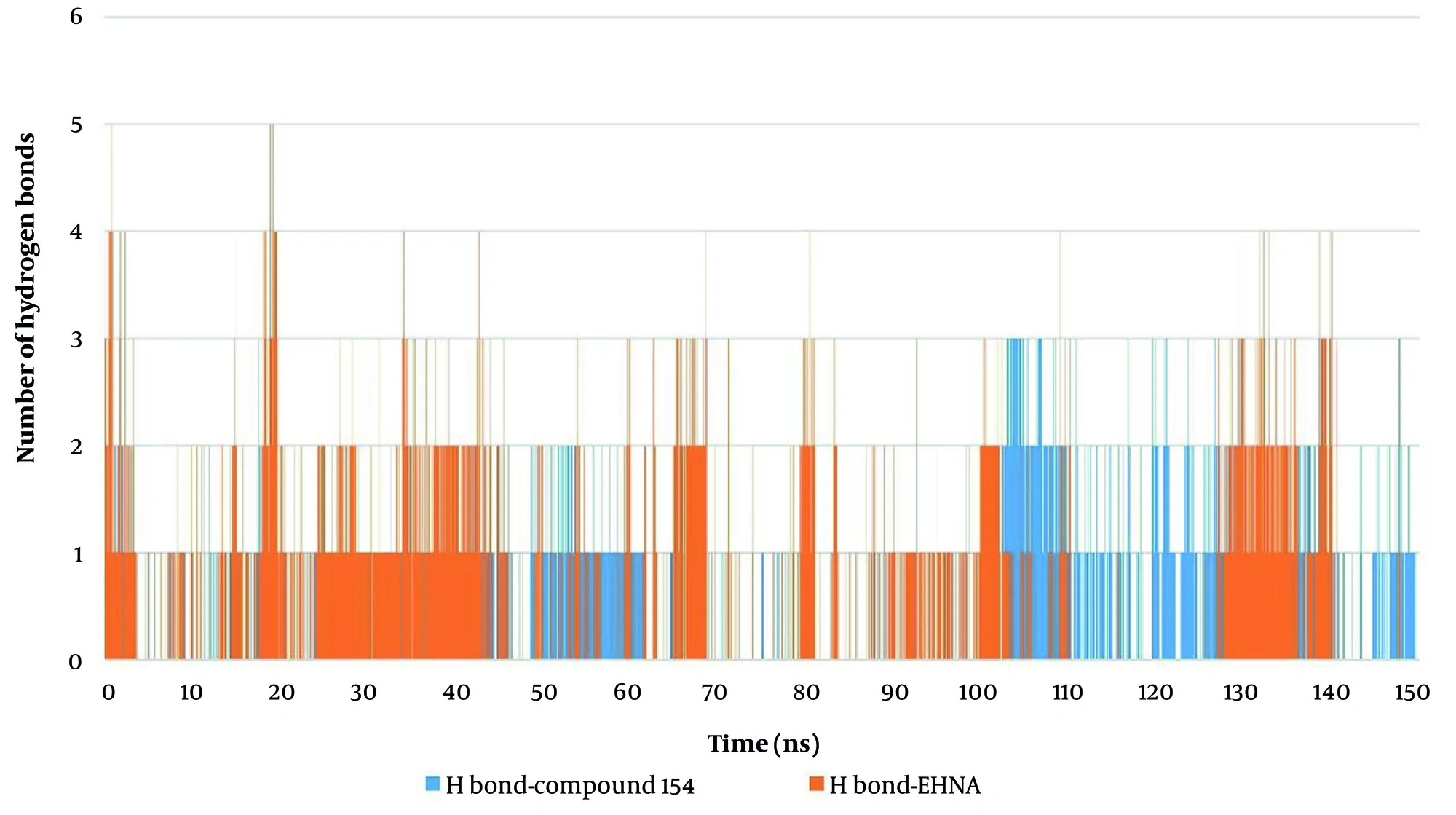
Number of hydrogen bonds formed between ADA and ligands, compound 154 (blue) and EHNA as the reference ligand (orange), during the 150 ns molecular dynamics simulation.

The stability of ADA–ligand interactions was further evaluated through hydrogen-bond analysis during the 150 ns MD simulation (Figure 8). The reference complex exhibited a slightly higher average number of hydrogen bonds (0.53) than the compound 154 complex (0.43); however, it also showed greater fluctuations and larger variations in hydrogen-bond occupancy. In contrast, compound 154 maintained a more consistent interaction profile, predominantly forming stable 1 - 2 hydrogen bonds throughout the simulation and demonstrating interaction stability during the final phase of the simulation (100 - 150 ns). These findings suggest that compound 154 achieves stable binding through specific and persistent hydrogen-bond interactions rather than through a greater number of highly variable contacts.

The ADA–compound 154 complex demonstrated favorable dynamic behavior characterized by lower protein RMSD and RMSF values, stable compactness (Rg), and more persistent hydrogen-bond interactions, supporting enhanced structural stability and reliable binding throughout the 150 ns simulation.

### 4.5. MM-GBSA and Per-Residue Decomposition Energy Calculations

#### 4.5.1. MM-GBSA Binding Free Energy Calculation

The binding free energy between 2Z7G and compound 154 was calculated. The MM-GBSA decomposition analysis is summarized in Table 4.

**Table 4.** Calculated Binding Free Energy (kcal/mol) from MM-GBSA ^a^

Energy Term	Average (kcal/mol)
**ΔE_VDWAALS_**	-15.12 ± 6.38
**ΔE_EL_**	-12.93 ± 10.47
**ΔG_GB_**	20.35 ± 10.64
**ΔG_SURF_**	-2.19 ± 0.91
**ΔG_GAS_**	-28.05 ± 14.37
**ΔG_SOLV_**	18.16 ± 9.96
**ΔG_bind_**	-9.89 ± 5.26

^a^ Abbreviations: Δ_VDWAALS_, van der Waals; ΔE_EL_, electrostatic; ΔG_GB_, polar solvation; ΔG_SURF_, nonpolar solvation; ΔG_GAS_, gas phase; ΔG_SOLV_, solvation.

The total binding free energy (ΔG_bind_) was calculated as −9.89 ± 5.26 kcal/mol, indicating favorable binding of compound 154 within the ADA active site. Van der Waals interactions (ΔE_VDWAALS_ = −15.12 ± 6.38 kcal/mol) and electrostatic interactions (ΔE_EL_ = −12.93 ± 10.47 kcal/mol) contributed favorably to ligand binding, whereas solvation energy (ΔG_SOLV_) was unfavorable, with a value of 18.16 ± 9.96 kcal/mol, primarily because of positive polar solvation energy (ΔG_GB_) of 20.35 kcal/mol.

The MM-GBSA results suggest that hydrophobic and electrostatic interactions are the driving forces stabilizing the ADA–compound 154 complex despite unfavorable polar solvation. This finding is consistent with the docking and MD analyses.

#### 4.5.2. Per-Residue Decomposition Energy Calculations

Per-residue energy decomposition analysis revealed several key amino acid residues contributing to compound 154 binding affinity (Table 5). Residues MET152, ASP293, and PHE297 showed negative energy values, indicating favorable contributions to ligand binding and suggesting roles in stabilizing compound 154 within the ADA binding pocket. The decomposition analysis further demonstrated that both electrostatic and van der Waals interactions contribute to the binding stability of compound 154.

**Table 5.** Energetic Decomposition (kcal/mol) Results for Residues of Interest

Residue	ΔE_VDWAALS_	ΔE_EL_	ΔG_GB_	ΔG_SURF_	ΔG_bind_
**ALA:180**	-0.00008	-0.00188	0.00198	-	0.00003
**ASP:292**	-0.00219	0.11045	-0.09903	-	0.00922
**ASP:293**	-0.00840	0.10265	-0.10580	-	-0.01155
**GLU:214**	-0.00009	0.04700	-0.04519	-	0.00172
**GLY:181**	-0.00005	0.00042	-0.00030	-	0.00007
**HIS:14**	-0.00241	0.02727	-0.02440	-	0.00046
**HIS:235**	-0.00018	0.00407	-0.00245	-	0.00144
**LEU:55**	-0.00019	0.00058	-0.00016	-	0.00022
**LEU:59**	-0.00039	0.00151	-0.00051	-	0.00061
**MET:152**	-0.00010	-0.00084	0.00085	-	-0.00009
**PHE:297**	-0.01782	-0.00700	0.02396	-	-0.00087
**PHE:58**	-0.00111	0.00029	0.00269	-	0.00187
**PHE:62**	-0.00206	-0.00068	0.00834	-	0.00560
**SER:262**	-0.00113	-0.00923	0.01181	-	0.00146
**THR:266**	-0.00045	-0.00290	0.00436	-	0.00101
**TRP:114**	-0.00065	-0.00180	0.00568	-	0.00323

### 4.6. In Silico Physicochemical and ADMET Properties

The physicochemical, ADMET, and drug-likeness properties of compound 154, the selected hit compound, along with EHNA and pentostatin as reference ADA inhibitors, were calculated using the ADMETlab 3.0 server (Table 6).

**Table 6.** Physicochemical, ADMET, and Drug-Likeness Properties of Selected Compounds ^a^

Properties and Parameters	Compound 154	EHNA	Pentostatin
**Physicochemical Properties**			
Formula	C_16_H_13_NO_2_	C_14_H_23_N_5_O	C_11_H_16_N_4_O_4_
Molecular weight (g/mol)	251.09	277.19	268.12
nHA	3	6	8
nHD	2	3	4
TPSA (Å^2^)	49.33	89.85	112.13
**Lipophilicity**			
Log P	2.568	2.061	-1.451
**Drug likeness**			
Lipinski Rule	Yes; 0 violations	Yes; 0 violations	Yes; 0 violations
**Medicinal Chemistry**			
Synthetic accessibility score ^b^	1 (easy)	3 (easy)	4 (easy)
**Pharmacokinetics**			
HIA	Excellent	Excellent	Excellent
P-gp substrate	No	No	Yes
CYP1A2 inhibitor	Yes	No	No
CYP2C19 inhibitor	No	No	No
CYP2C9 inhibitor	No	No	No
CYP2D6 inhibitor	No	No	No
CYP3A4 inhibitor	Medium	Yes	No
**Toxicology ^c^**			
Genotoxicity	0.958	0.896	0.999
Carcinogenicity	0.675	0.701	0.748
AMES mutagenicity	0.616	0.643	0.853
A549 cytotoxicity	0.031	0.328	0.127
HEK293 cytotoxicity	0.264	0.487	0.139
Human hepatotoxicity	0.888	0.818	0.883
Skin sensitization	0.863	0.523	0.993
Drug-induced nephrotoxicity	0.715	0.670	0.892

^a^ Abbreviations: nHA, number of H-bond acceptors; nHD, number of H-bond donors; TPSA, topological polar surface area; logP, logarithm of the n-octanol/water distribution coefficients at pH 7.4; molecular weight ≤ 500 g/mol, logP ≤ 5, nHA ≤ 10, nHD ≤ 5; HIA, human intestinal absorption.

^b^ Score ≥ 6, difficult to synthesize; score < 6, easy to synthesize;

^c^ The output value is the probability of being toxic.

The physicochemical properties and drug-likeness of the three compounds were compared. Compound 154 was the most lipophilic compound (LogP = 2.568) and exhibited the lowest TPSA (49.33 Å^2^), suggesting favorable membrane permeability and passive diffusion. All three compounds obeyed the Lipinski rule of five with no violations, suggesting favorable oral drug-likeness. The synthetic accessibility scores also indicated that compound 154 is not difficult to synthesize.

All three compounds demonstrated appropriate predicted human intestinal absorption. Because compound 154 was predicted to be a non-substrate of P-glycoprotein, it may exhibit a favorable absorption profile. In addition, compound 154 showed inhibitory potential toward CYP1A2 and CYP3A4, whereas no significant inhibitory effects were predicted for CYP2C9, CYP2C19, and CYP2D6.

Based on the predicted toxicity parameters, compound 154, similar to EHNA and pentostatin, demonstrated high probabilities of genotoxicity and hepatotoxicity, along with moderate carcinogenicity. In addition, compound 154 showed predicted probabilities of AMES mutagenicity, skin sensitization, and drug-induced nephrotoxicity comparable to those predicted for the reference inhibitors. In contrast, the predicted probability values for toxicity toward A549 (human lung cell line) and HEK293 (human embryonic kidney cell line) were low, suggesting limited cytotoxic potential against these cell lines. Compound 154 demonstrated suitable physicochemical and pharmacokinetic properties; however, its predicted toxicity profile indicates that further structural optimization and experimental toxicity evaluations are required.

## 5. Conclusions

In this study, a pharmacophore model for ADA inhibitors was developed and validated by combining structural features from two co-crystallized ligands, EHNA and pentostatin. The resulting shared-feature pharmacophore model was reliable and selective, as confirmed by ROC curve analysis, the enrichment factor, and performance.

Screening the in-house library identified four hits. Docking studies showed that compound 154 exhibited suitable binding affinities for both crystal structures (PDB IDs: 2Z7G and 2PGR). The docking results were consistent with the pharmacophore screening outcomes. Furthermore, MD simulations showed that the ADA–compound 154 complex remained stable over a 150 ns trajectory. MM-GBSA free energy calculations and per-residue decomposition indicated that binding was primarily driven by van der Waals and electrostatic interactions.

In silico physicochemical and ADMET evaluations demonstrated that compound 154 has favorable drug-likeness and synthetic accessibility. However, the predicted toxicity profile suggests potential toxicity that may limit its therapeutic applicability without further structural optimization.

Compound 154 appears to be a promising hit candidate; however, additional in vitro and in vivo studies are required to validate its biological activity and safety profile. Future studies should focus on expanding chemical structure screening, improving pharmacokinetic and toxicity profiles, and exploring structure–activity relationships to optimize the hit.

Overall, these results indicate that pharmacophore modeling is an effective approach for discovering ADA inhibitors.

## Data Availability

The data presented in this study are available upon request
